# Primary Appendiceal Adenocarcinoma Masquerading as Acute Appendicitis: A Case Report

**DOI:** 10.7759/cureus.28903

**Published:** 2022-09-07

**Authors:** Grace Young, Zachary Brennan, Rafael Figueroa

**Affiliations:** 1 Surgery, A.T. Still University - Kirksville College of Osteopathic Medicine, Kirksville, USA; 2 Surgery, Michigan State University College of Osteopathic Medicine, East Lansing, USA; 3 Colorectal Surgery, Parkland Health Center, Farmington, USA

**Keywords:** hemicolectomy, appendectomy, appendiceal carcinoma, general surgery, appendicitis

## Abstract

Primary adenocarcinoma of the appendix is a rare neoplasm, and a large number are found incidentally during abdominal imaging and operations with other indications. This report examines a case of primary appendiceal adenocarcinoma discovered incidentally following a seemingly routine laparoscopic appendectomy in a 69-year-old male. The patient presented with atypical signs of acute appendicitis, including a history of waxing and waning right lower quadrant pain without anorexia, fever, or chills over five days. After undergoing laparoscopic appendectomy, histopathologic analysis of the appendix specimen revealed invasive adenocarcinoma. This case emphasizes the importance of maintaining a high index of suspicion for appendicitis versus appendiceal neoplasm in older adults presenting with atypical signs and symptoms.

## Introduction

Appendectomy for acute appendicitis is the most performed surgery in the United States, with approximately 300,000 operations performed each year [1]. Of the specimens sent to pathology following these operations, appendiceal malignancy accounts for only 0.5%-1% [2]. Epithelial neoplasms are seen in 0.2%-0.3% of appendectomy specimens, while neuroendocrine tumors are seen in 0.3%-0.9% of specimens [3]. While the pathophysiological mechanisms that result in primary adenocarcinomas of the appendix are not well understood, it has been hypothesized that they follow a similar adenoma-carcinoma sequence as seen in colorectal carcinoma [2]. An alternative hypothesis is that these malignancies occur secondary to microsatellite instability resulting from mutations in various mismatch repair genes [2].

The incidence of appendiceal neoplasms discovered incidentally during seemingly routine appendectomy has been found to rise with age, especially among adenocarcinoma subtypes with median ages of presentation in the fourth and fifth decades of life [1, 3-4]. The likelihood of finding neoplasms on histopathologic analysis of appendiceal specimens was significantly higher in complicated cases of appendicitis (12.6%) compared to uncomplicated cases (1.2%) [4]. The probability of neoplastic findings was also associated with increasing age at presentation, with those under 30 years of age having nearly 0% risk of appendiceal neoplasm, those between 39 and 50 years of age having an 11% risk, and those greater than 50 years of age having a 16% risk of a concealed neoplasm [4]. The mean age of diagnosis also varied significantly based on the type of neoplasm seen, with neuroendocrine tumors presenting in the fourth decade of life on average and adenocarcinomas presenting in the seventh decade on average [5].

## Case presentation

A 69-year-old Caucasian male presented to his local rural emergency department with right lower quadrant abdominal pain. He described the pain as a constant waxing and waning, non-radiating, cramping pain that had persisted for over five days. Pertinent negatives included no anorexia, chest pain, fever, chills, vomiting, or diarrhea. His last bowel movement was four days before his presentation.

On room air, the patient was afebrile with stable vital signs, including a temperature of 37.1C, pulse rate of 48 beats per minute, breath rate of 16 breaths per minute, blood pressure of 127/63, and oxygen saturation of 96%. Further examination showed a well-developed male in no acute distress. The abdomen was soft with tenderness in the right lower quadrant without guarding or rebounding. The exam was remarkable for right costovertebral area tenderness. He was negative for Murphy's sign, Rovsing's sign, McBurney's point, and psoas and obturator signs. A complete blood count with differential was unremarkable, as was a complete metabolic panel done at that time.

With these nonspecific findings, the differential diagnosis at this point was quite broad, including but not limited to appendicitis, nephrolithiasis, infectious colitis, inflammatory bowel disease, ischemic colitis, constipation, or irritable bowel syndrome.

**Figure 1:**
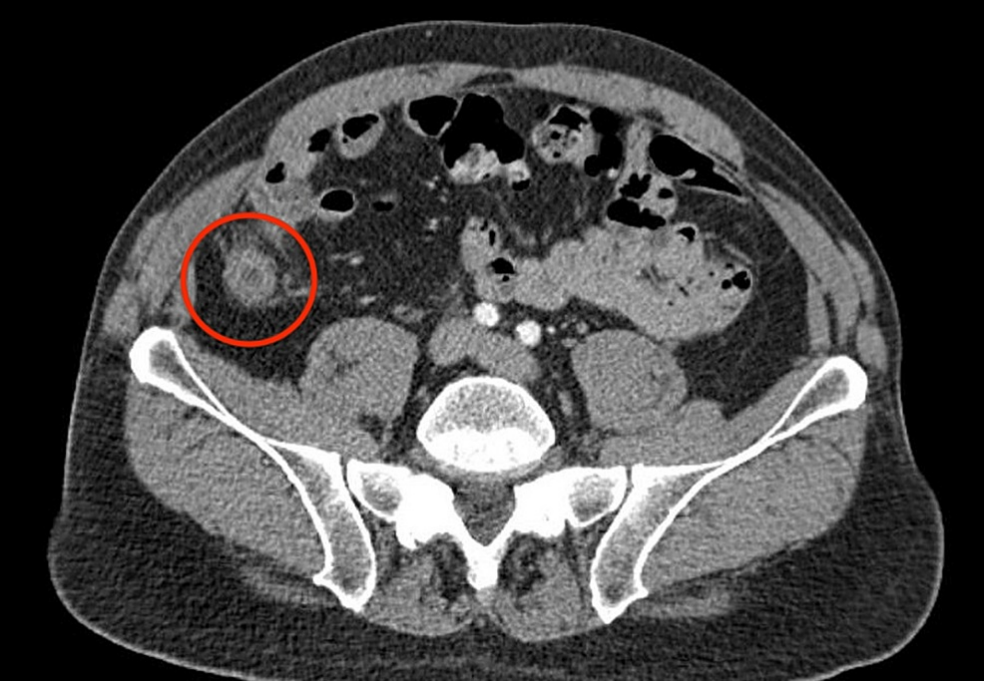
CT scan image (transverse view) showing appendiceal wall thickening.

**Figure 2:**
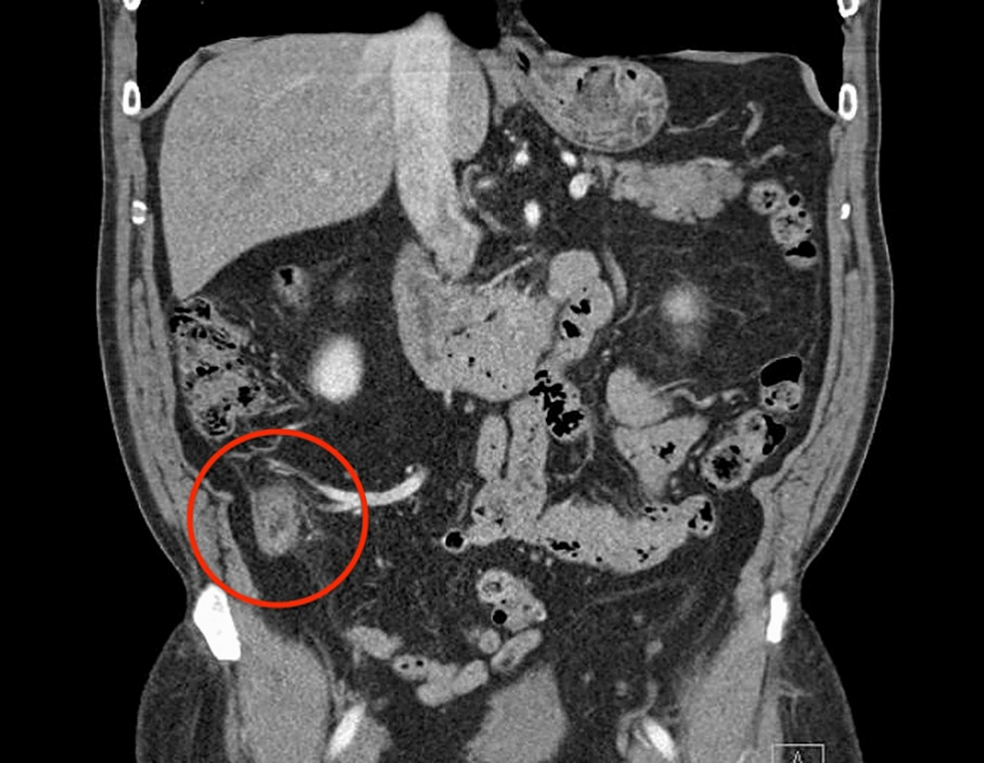
CT image (coronal View) showing appendiceal wall thickening.

The patient then underwent computed tomography (CT) imaging of the abdomen, which showed a markedly enlarged and thick-walled appendix with sections measuring up to 18 mm in diameter. A periappendiceal edema without extraluminal fluid, gas, or fluid collections was also visualized (Figure 1 and Figure 2).

The patient was taken to the operating room the following day to undergo a routine laparoscopic appendectomy. The procedure was without complication. The appendix showed signs of inflammation but appeared to be intact. The specimen and mesoappendix were placed in formalin solution and sent to pathology.

Surgical pathology showed moderately differentiated, enteric-type invasive adenocarcinoma involving the entire appendiceal lumen with invasion into the muscularis propria. The proximal resection margin was positive for carcinoma. As no other specimens were taken during the initial surgery, the pathologic stage was reported at pT2 pNx pMx. Immunohistochemical analysis was performed and showed no loss of MLH1, PMS2, MSH2, or MSH6 mismatch repair proteins, lowering the risk of neoplasm secondary to Lynch syndrome.

The patient was scheduled for a diagnostic colonoscopy three weeks after a diagnosis that showed non-bleeding internal hemorrhoids and diverticulosis in the sigmoid colon. No polyps or suspicious lesions were noticed, and no specimens were collected during the procedure. An additional review of the CT scan performed at the time of admission at the emergency department showed no sign of metastatic disease. The patient was cleared for laparoscopic right hemicolectomy with ileocolic anastomosis five days later as planned. No evidence of carcinomatosis or metastatic disease was noted upon visualization during the operation.

The postoperative course was complicated by a fever of 39.4C on day one after surgery and atelectasis on the chest X-ray. The fever resolved spontaneously within 24 hours. The patient had a partially liquid bowel movement without hematochezia on day three. He was without nausea, vomiting, fever, or chills, and was tolerating food by mouth without issue. On day three, he was discharged.

The pathology report found no residual carcinoma or high-grade dysplasia in the cecum or ascending colon, and both proximal and distal margins of resection were negative for tumor. Twenty-four lymph nodes were submitted for examination, and all were negative for malignancy. Incorporating the previous appendectomy case, the carcinoma was pathologically staged as pT2 pN0 pM0, American Joint Committee on Cancer (AJCC) Stage I. The operation was assumed to be curative, and there were no indications for chemotherapy. The patient was seen in the clinic for a follow-up two weeks after the operation. At that appointment, he was eating a regular diet without difficulty, and his bowel movements were solid but soft. He was in no pain. He was sent home with instructions to return one year later for a surveillance colonoscopy and computed tomography scan of the chest, abdomen, and pelvis.

## Discussion

This patient presented with very nonspecific signs and symptoms. His intermittent abdominal pain in the absence of anorexia, nausea, vomiting, fever, and chills, along with a negative obturator, Rovsing, and McBurney’s signs, gave little clinical suspicion of acute appendicitis. With positive imaging and limited clinical evidence, some providers may have found this patient to be an ideal candidate for non-operative management and treatment with antibiotic therapy. In this case, it is unknown how long it would have been before the patient’s neoplasm was discovered and treatment initiated. This case raises the question of non-operative management for older adults presenting with uncomplicated acute appendicitis, a population known to have an increased incidence of incidental neoplastic findings [1].

Appendiceal neoplasms often present as acute appendicitis following obstruction of the appendiceal lumen and are then discovered incidentally following appendectomy [2]. Because of this, it is important to maintain an index of suspicion for neoplasms in populations at higher risk prior to making the decision to pursue non-operative treatment. Several trials have been conducted to examine the efficacy of non-operative management in cases of acute appendicitis. The risk of recurrence grew with increased time from the initial presentation: at presentation, 10% of cases required appendectomy following management with antibiotics alone; within 90 days, 29%; within one year, 27.3%; and within five years, 39.1% of these cases required definitive operative management [4].

According to the Multicenter Study of the Treatment of Appendicitis in America: acute, perforated, and gangrenous (MUSTANG) trial, the incidence of neoplasm was highest in patients greater than 40 years of age and with an appendiceal diameter of greater than one centimeter on a CT scan, both of which were seen in this patient [1]. Another significant study, the Peri-Appendicitis Acute (periAPPAC) trial, published in 2019, examined patients between the ages of 18 and 60 with appendiceal abscesses treated with antibiotic therapy [4]. This trial had to be terminated early after it was discovered that 17% of all of those enrolled were found to have an appendiceal neoplasm; in those over 40 years of age, the rate of neoplasm was 35% [4]. Another study discovered that one in every five people who were initially treated non-operatively required an appendectomy within the next 30 days [6].

With a clinical presentation including several non-specific symptoms and pertinent negatives, including lack of fever, chills, or anorexia, along with an unremarkable complete blood count and positive CT scan, this patient may have been an ideal candidate for non-operative management. An urgent appendectomy with rapid diagnosis is critical for appendiceal neoplasms that present early with atypical appendicitis symptoms. In patients over 40 years of age presenting with appendicitis, the index of suspicion for neoplasms should be higher and the modality of treatment appropriately determined [1].

## Conclusions

While malignancy is a rare cause of appendicitis, many cases arise each year. When these older patients present for appendicitis, malignancy should always be on the differential. This is especially true when patients present with atypical signs and symptoms of appendicitis, as with this case. Though non-operative treatment is a growing modality for atypical appendicitis, it is important to take patient age and other risk factors for neoplasms into consideration when making this decision. Surgeons and emergency room physicians should be aware of malignancy as a potential etiology of appendicitis.
